#  Effect of Jingqian Zhitong Fang on Serum Sex Hormone Levels in Women with Primary Dysmenorrhea

**DOI:** 10.1155/2014/876431

**Published:** 2014-04-16

**Authors:** Na Dai, Ling Fang, Yu-bo Li, Yu-ming Wang, Ju Yin, Bao-chan Pu

**Affiliations:** ^1^Tianjin State Key Laboratory of Modern Chinese Medicine, School of Traditional Chinese Materia Medica, Tianjin University of Traditional Chinese Medicine, No. 312 Anshan West Road, Tianjin 300193, China; ^2^Obstetrics and Gynecology Hospital in Tianjin, Tianjin 300193, China

## Abstract

Primary dysmenorrhea is a common gynecological disease garnering increasing attention and research. To investigate the clinical therapeutic effects of Jingqian Zhitong Fang (JQF) and the differences in serum sex hormone levels during the treatment of primary dysmenorrhea, we selected 30 healthy volunteers and 60 individuals with primary dysmenorrhea. On the third day of the menstrual cycle, we used ELISA to determine the levels of serum prolactin (PRL), follicle-stimulating hormone (FSH), luteinizing hormone (LH), testosterone (TEST), progesterone (PROG), and estradiol (E_2_) compared with normal levels and levels in the JQF group, the Western medicine group receiving continuous treatment during the first and third menstrual cycles, and the group followed up after the drug was stopped. We observed that after JQF treatment, the levels of the following hormones changed significantly: PRL, LH, TEST, and E_2_ levels decreased significantly and the PROG level increased significantly after treatment. After treatment with Western medicine, the serum levels of FSH, LH, PROG, and E_2_ showed no significant change. We conclude that the long-term effect of JQF treatment was better than that of Western medicine. JQF treatment of primary dysmenorrhea is related to adjustment of PRL, LH, TEST, and E2 hormone levels in the human body.

## 1. Introduction

Primary dysmenorrhea (PD) is characterized by cramping pelvic pain at the onset of menses, lasting for 48–72 hours during the menstrual cycle, and is highly prevalent (approximately 50%) among adolescent girls [1, 2]. According to an epidemiological sampling survey conducted in 1980, primary dysmenorrhea is a common gynecological disease. In China, the dysmenorrhea incidence was 33.1%, with primary dysmenorrhea patients accounting for 53.2% and dysmenorrhea patients heavily influenced by the work accounting for 13.55% [3]. Because of dysmenorrhea, six-hundred million work days are lost annually in the USA, leading to economic losses of up to 200 million dollars [4].

The hormonal characteristics of humans have been reported. In the menstrual cycle, prostaglandins are smooth muscle stimulants [5]. In the luteal phase, the endometrium produces more prostaglandins. The PGE_2_ content is higher than PGF_2*α*_ content, which is opposite to that during the menstrual cycle. Powell et al. found that the levels of menstrual PGF_2*α*_ and PGE_2_ were significantly higher in women with dysmenorrhea than in healthy women [6]. The serum levels of estradiol (E_2_), luteinizing hormone (LH), and follicle-stimulating hormone (FSH) play a similar role in women with dysmenorrhea [7]. The abnormal hormone levels caused by primary dysmenorrhea can cause a variety of diseases. Progesterone (PROG) is a major progestational steroid that can decrease the contractility of uterine smooth muscle. High progesterone levels can decrease the incidence of breast cancer. In contrast, they can cause tumor formation [8]. Estradiol is an estrogen whose catechol metabolites that bind with proteins and nucleic acids can lead to cell damage and induce cancer [9]. Some studies have reported that primary dysmenorrhea may be caused by endometriosis, which can aggravate dysmenorrhea [10]. Patients with both adenomyosis and endometriosis show high levels of E_2_, FSH, and LH [7]. Therefore, it is necessary to study the relationship between the occurrence of dysmenorrhea and the disequilibrium of sex hormones.

The Chinese herbal formula Jingqian Zhitong Fang (JQF) is considered to be an effective prescription for the treatment of PD. JQF originally came from Foshou san, which, in clinical practice, has been proven to treat cold retention and blood in gynecological diseases such as primary dysmenorrhea. Angelica is the main drug in JQF and could regulate menstrual pain. Angelica oil (including 70% ligustilide), regardless of whether the uterus is normal and nonpregnant or has been treated with oxytocin, can relieve uterine smooth muscle pain [11]. Motherwort can regulate menstruation [12]. Chuanxiong was used in the clinical treatment of irregular menstruation, amenorrhea dysmenorrhea, and headache. And its main components: ferulic acid, tetramethylpyrazine, and ligustilide have an antispasmodic effect on smooth muscles and can lead to uterine smooth muscle relaxation [13, 14]. Radix paeoniae Rubra is rich in glycosides, which can heat and cool blood, heal bruises, and relieve pain [15]. On the basis of experimental pharmacological studies, JQF can significantly inhibit oxytocin-induced cramping reflexes in mice and can also increase pain thresholds [16].

In this study, we collected plasma samples from the PD patients and volunteers and used ELISA to compare the different levels of six serum sex hormones. At the same time, we found variations in the changes in every sex hormone during treatment. After treatment, we compared the sex hormone levels between PD patients (JQF treatment group and Western medicine group) and healthy volunteers to determine whether the differences were important indicators of the clinical efficacy of JQF.

## 2. Materials and Methods

### 2.1. Study Population and Design

The protocol for this study was reviewed and approved by Tianjin University of Traditional Chinese Medicine. From January 2010 to December 2013, 30 volunteers without dysmenorrhea (age, 16–28 years; mean, 21.66 ± 3.03 years) and with body weight of 42–69 kg (mean, 56.81 ± 8.62 kg) were selected. Sixty outpatients with primary dysmenorrhea were identified in the Obstetrics and Gynecology Hospital in Tianjin and Affiliated HealthCare Hospital of Tianjin University of Traditional Chinese Medicine and were randomly divided into the JQF treatment group and the Western medicine group. Those in the JQF group were aged 15–32 years old (mean, 21.33 ± 4.27 years) with the course of disease lasting from 1 to 14 years (average, 6.60 ± 3.81 years) and mean body weight of 58.69 ± 8.00 kg. Those in the Western medicine group were aged 14 to 30 years (mean, 21.42 ± 5.32 years) with mean body weight of 58.48 ± 9.25 kg and a course of disease lasting from 1.5 to 15 years (average, 6.64 ± 3.70). All primary dysmenorrhea patients were nonpregnant and showed no abnormalities upon gynecological examination. In the corpus luteum atrophy period, continuous treatment was administered during three menstrual cycles, with curative effects evaluated for the first menstrual cycle and the third menstrual cycle, and follow-up. At all time points, menstrual blood was collected on the third day of the menstrual cycle for detection of the levels of six serum sex hormones.

### 2.2. Drug Administration

JQF includes, among other components,* Angelica sinensis*,* Rhizoma chuanxiong*, and* Radix paeoniae Rubra* (confirmed by the Tianjin University of Traditional Chinese Medicine Plants Laboratory). To avoid possible biases resulting from differences in herbs produced in different areas, all necessary herbs were purchased together. The steps in JQF medication preparation are as follows: the herbs are mixed together according to their prescribed dosages, and water 10 times the volume of the herbs is added, with the mixture being boiled for 1 h. The first extract is poured, the same volume of water is added, and the mixture is boiled for another hour. The second extract is poured and the two extracts are mixed. Finally, after concentration and drying, a solid extract is obtained. The ratio of solid extract to the herbs' dry weight was 24.6%. Quantitative analysis of the chemical composition of JQF has been performed (see Supplementary Text 1 in Supplementary Material available online at http://dx.doi.org/10.1155/2014/876431). The primary dysmenorrhea group received doses of JQF or oral ibuprofen during the corpus luteum atrophy period (3–5 days before menstruation) with continuous treatment during three menstrual cycles. Dosage was discontinued in the fourth cycle.

### 2.3. Sample Preparation Detection Method

Blood samples were taken from volunteers on the third day of the menstrual cycle, placed in a water bath at 30°C for 30 min then centrifuged at 3600 g for 10 min. The supernatant was injected into the ELISA kit and used for the quantitative determination of six serum sex hormone levels (PRL, FSH, LH, TEST, PROG, and E_2_) on the third day of the menstrual cycle. According to the hypothalamic-pituitary-ovarian axis changes in the menstrual cycle, in the first to seventh days of menstruation, the levels of PRL, FSH, LH, PROG, and E_2_ showed little fluctuation, which was easy to manipulate, so venous blood was collected on the third day of the menstrual cycle.

### 2.4. Efficacy Criteria

According to the Guiding Principles of Clinical Research on Traditional Chinese Medicine for new drugs, issued by the Ministry of Health, People's Republic of China, we established the following dysmenorrhea scoring criteria: mild, dysmenorrhea symptom score of <8 points; moderate, dysmenorrhea symptom score from 8 to 13.5 points; and severe, dysmenorrhea symptom score of >14 points.

The posttreatment efficacy criteria were as follows: the dysmenorrhea symptoms disappeared or were significantly decreased, and comparison with pretreatment of dysmenorrhea symptoms showed a marked reduction of >50% in the symptom score; the symptoms of dysmenorrhea were alleviated, and the dysmenorrhea symptom score was effectively decreased by 25%–50%; or there was no obvious improvement in dysmenorrhea and other symptoms, with the dysmenorrhea symptom score decreased by <25%, thus rendering treatment ineffective.

### 2.5. Statistical Analyses

The measurement data were expressed as means ± standard deviation (*χ* ^2^ ± *S*), with analysis of variance and correlation tests. The *χ* ^2^ test was used for counting data, with *α* = 0.05, and data were analyzed with SPSS11.5 software. The correlation tests were analyzed with SIMCA 11.5 software.

## 3. Results

The results after JQF treatment for 1 month, 3 months, and follow-up showed that the total clinically effective rates were 88.7%, 94.7%, and 94.1%, respectively (Table 1). The dysmenorrhea symptom score decreased after treatment. Effects were weak after treatment for the “one menstrual cycle” group (*P* < 0.05), but there were significant effects of treatment for the “three menstrual cycles” and follow-up groups (*P* < 0.01) (Table 2). Venous blood was collected on the third day of the menstrual cycle, and the six serum sex hormone levels were measured. The results showed that the PRL, TEST, and E_2_ levels were higher in the dysmenorrhea group than in the healthy groups (*P* < 0.01). After treatment for one or three menstrual cycles, the three serum sex hormone levels were lower than those in the “no treatment” group (*P* < 0.01) and were almost the same as those in the healthy group. The FSH levels did not differ among all the groups (dysmenorrhea model group, treatment group, follow-up group, and the healthy group) (*P* > 0.05). Compared with the normal group, the LH level in the dysmenorrhea group increased (*P* < 0.05). There was no significant difference (*P* > 0.05) between the “one menstrual cycle” and dysmenorrhea groups, but the LH level decreased significantly in the “three menstrual cycles” and follow-up groups (*P* < 0.05). Compared with the normal group, the PROG level showed a downward trend in the dysmenorrhea group; however, after treatment, it expressed an upward trend in all the treatment groups (Table 3).

The total clinically effective rates at 1 month, 3 months, and follow-up after Western medicine treatment were 93.3%, 90.0%, and 20.0%, respectively. (Table 1) The dysmenorrhea symptom scores were significantly decreased in the “one menstrual cycle” and “three menstrual cycles” groups (*P* < 0.01); however, there was no difference between the pretreatment group and the follow-up group (Table 2). Compared with those in normal volunteers, the PRL, LH, TEST, and E_2_ levels were higher in the dysmenorrhea patients before treatment (*P* < 0.01). After Western medicine treatment for the first and third menstrual cycles, the four hormone levels were decreased (*P* < 0.05). However, during follow-up, we found that LH and E_2_ had returned to pretreatment levels. There was no difference in FSH and PROG in the different groups. Western medicine treatment had a rapid onset of features, but the results from the follow-up showed that its long-term prognosis was poor.

**Figure 1:**
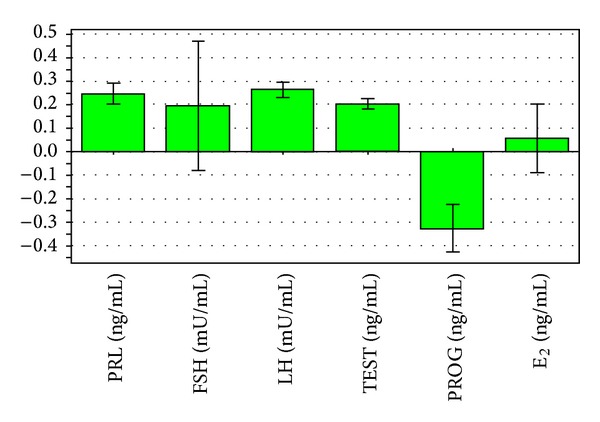
The correlation coefficients between the levels of six hormones and the scores for the extent of the disease (the bars indicate the confidence intervals of the coefficients. The coefficient is significant when the confidence interval does not cross zero.).

The levels of six serum hormones in a certain time period have the combined effect of the degree of dysmenorrhea integration. Therefore, by correlation analysis with six kinds of hormone levels and dysmenorrhea integrals, we have established a regression equation: *Y* = 1.15125 + 0.24804*X*_1_ + 0.19443*X*_2_ + 0.26509*X*_3_ + 0.20469*X*_4_ − 0.32597*X*_5_ + 0.05783*X*_6_ (*Y*, dysmenorrhea inregrals; *X*_1_, PRL; *X*_2_, FSH; *X*_3_, LH; *X*_4_, TEST; *X*_5_, PROG; *X*_6_, E_2_). PRL, FSH, LH, TEST, and E_2_ positively associated with dysmenorrhea integrals and PROG negatively correlated with dysmenorrhea integral. The PRL, PROG, LH, and TEST have a larger impact of dysmenorrhea integral than E_2_ and FSH (Figure 1).

## 4. Discussion

JQF includes, among other components,* Angelica sinensis*,* Rhizoma chuanxiong*, and* Radix paeoniae Rubra*.* Angelicae sinensis* can enrich, supplement, and nourish blood.* Rhizoma chuanxiong* is regarded as a component that can result in improvements in the respiratory and cardiovascular systems. The complete prescription prevents diarrhea and results in improvement of the cardiovascular and respiratory systems.

Ibuprofen has analgesic, antipyretic, and anti-inflammatory effects and is one of a group of nonsteroidal anti-inflammatory analgesics. Because it has a certain stimulus in the gastrointestinal tract, it can cause relative reduction of the side-effects of sustained-release formulations and can allow for the gradual release of drugs in the body. Ibuprofen sustained-release capsules are a prostaglandin synthetase inhibitor and can decrease the production of prostaglandins to prevent uterine contractions and spasms, thereby decreasing or eliminating dysmenorrhea. Their effects are rapid, and adverse reactions are mild. However, there are disadvantages in that symptoms can easily recur after treatment is discontinued.

The results of this study showed that, before and after treatment, the total efficiency and dysmenorrhea symptom scores of the JQF therapy group and Western medicine group showed significant differences in one cycle and three cycles. At follow-up, the patients were significantly better after JQF treatment than after Western medicine treatment, and there was a significant difference between the two groups.

Analysis of biochemical data showed that the serum endocrine index was significantly changed (*P* < 0.05) after JQF treatment. The PRL, LH, TEST, and E_2_ levels were significantly decreased, whereas the PROG levels were significantly increased after treatment. These data are consistent with our previous explore on trends of JQF treatment in dysmenorrhea animal models on the levels of E_2_ and P. Further validated, JQF had a good therapeutic effect on primary dysmenorrhea (Table 4). It also showed an overall efficiency in regulating the biochemical parameters related to endocrinology [17, 18]. On JQF group, 11 patients were excluded in the third menstrual cycle, because they refused to take the medications or suffer from diseases. Sixteen patients were treated effectively. The integral in the fourth menstrual cycle was 1.43 ± 1.12 in the follow-up period. Compared with the first menstrual cycle (7.35 ± 3.15), the integral of the symptoms of dysmenorrhea was significantly decreased.

The luteal estrogen levels were significantly higher in the test group than in the control group, suggesting that the abnormal synthesis of endometrial PGs may be related to excessive estrogen [19]. The increased E_2_ level may have indirectly promoted the synthesis and release of PGF_2*α*_, resulting in uterine contractions and uterine ischemia or angiospasm, causing dysmenorrhea (Table 4). The endometrium can also produce estrogen, and high estrogen levels promote excessive uterine contractions and cause diseases [20]. Our study shows that the level of estrogen is positively correlated with dysmenorrhea. PROG is antagonistic to E_2_, meaning that the increasing content of PROG inhibits the production of PGF_2*α*_ to relieve uterine smooth muscle spasms. Such antagonism can also trigger dysmenorrhea indirectly via affecting the synthesis and release of other hormones, such as oxytocin, vasopressin, and endogenous opioid peptides [21].

## 5. Conclusions

In conclusion, estrogen can promote the pituitary to secrete PRL and inhibit the secretion of dopamine. Therefore, we can decrease DA levels to relieve the inhibition of PRL, whereas estrogen levels are high. Androgens, especially testosterone, can promote PRL secretion. LH subunits can cause increased PRL secretion. In the menstrual cycle, the secretion of PRL showed no significant change. Peripheral blood with a concentration of PRL is one of the contributors to menstrual disorders.

The results showed that changes in PRL, LH, TEST, PROG, and E_2_ concentrations are correlated with dysmenorrhea.

## Supplementary Material

JQF quantitative analysis was performed using high performance liquid chromatography-triple quadrupole mass spectrometry (HPLC-QqQ-MS) in positive and negative ion modes. The separation was performed on an Eclipse plus C18, 4.6∗10 mm, 3.5 um, by gradient elution using 0.1% formic acid in water (mobile phase A) and 0.1% formic acid in acetonitrile as mobile phases. 13 peaks (stachydrine, gallic acid, oxypaeoniflorin, chlorogenic acid, rutin, paeoniflorin, caffeic acid, vanillic acid, ferulic acid, benzoyl paeoniflorin, senkyunolide A, and ligustilide) in the HPLC-MS chromatograms were unequivocally identified by comparison of their retention times (RTs), molecular weights, and MS data with reference data from the literature. Compound concentrations and percentages of ingredients in JQF are listed in Table S2 and Table S3.

## Figures and Tables

**Table 1:** Comparison of women with primary dysmenorrhea in the first and third menstrual cycles with JQF and Western medicine treatment and follow-up after treatment [*n* (%)].

	*n*	Markedly effective	Effective	Ineffective	Total efficiency
J one cycle	30	16 (53.3)	10 (33.3)	4 (13.3)	88.7
J three cycles	19	13 (68.4)	5 (26.3)	1 (5.3)	94.7
J follow-up	17	13 (76.5)	3 (17.6)	1 (5.9)	94.1
W one cycle	30	24 (80.0)	4 (13.3)	2 (6.7)	93.3
W three cycles	20	14 (70.0)	4 (20.0)	2 (10.0)	90.0
W follow-up	20	0 (0.0)	4 (20.0)	16 (80.0)	20.0

“W” stands for the Western medicine treatment group; “J” stands for the JQF treatment group.

**Table 2:** The integral contrasts in primary dysmenorrhea after treatment.

	JQF group (*n*)	Western medicine group (*n*)
Before treatment	11.52 ± 3.36 (30)	11.86 ± 2.98 (30)
The first cycle	7.35 ± 3.15* (30)	8.86 ± 2.76** (30)
The third cycle	1.86 ± 1.69** (19)	2.56 ± 1.54** (20)
Follow-up	1.43 ± 1.12** (17)	11.88 ± 4.13 (20)

**Integral values of the degree of dysmenorrhea before and after treatment, *P* < 0.01. *P < 0.05.

**Table 3:** Comparison of serum sex hormone levels in women with primary dysmenorrhea after JQF treatment and follow-up (*χ* ^2^ ± *S*).

	PRL (ng/mL)	FSH (mIU/mL)	LH (mIU/mL)	TEST (ng/mL)	PROG (ng/mL)	E_2_ (ng/mL)
Controls (*n* = 30)	13.93 ± 2.65	3.71 ± 0.64	6.64 ± 1.69	0.91 ± 0.16	1.14 ± 2.06	70.00 ± 13.57
J dysmenorrhea (*n* = 30)	19.70 ± 4.09^##^	3.68 ± 0.67	8.05 ± 3.64^#^	1.33 ± 0.23^##^	0.58 ± 0.20	99.80 ± 25.80^##^
J one cycle (*n* = 30)	15.75 ± 1.26**	3.95 ± 0.70	6.86 ± 3.06	1.01 ± 0.20**	0.77 ± 0.41*	67.85 ± 11.72**
J three cycles (*n* = 19)	13.58 ± 2.24**	3.78 ± 0.36	5.99 ± 0.90*	0.89 ± 0.12**	1.02 ± 0.46*	69.05 ± 8.40**
J follow-up (*n* = 17)	12.90 ± 2.41**	3.87 ± 0.51	6.00 ± 1.30*	0.87 ± 0.15**	1.04 ± 0.44**	68.23 ± 11.07**
W dysmenorrhea (*n* = 30)	21.56 ± 2.88^##^	4.31 ± 0.82	9.74 ± 2.42^#^	1.34 ± 0.16^##^	0.56 ± 0.19	104 ± 18.90^##^
W one cycle (*n* = 30)	19.13 ± 0.99*	3.93 ± 0.37	6.61 ± 1.28**	0.92 ± 0.11**	0.73 ± 0.16	85.75 ± 9.39**
W three cycles (*n* = 20)	18.6 ± 0.89*	4.02 ± 0.45	6.2 ± 0.77**	0.89 ± 0.08**	0.8 ± 0.13	86.57 ± 9.28*
W follow-up (*n* = 20)	19.27 ± 1.40*	3.74 ± 0.60	7.10 ± 0.60	1.02 ± 0.30**	0.71 ± 0.24	89.00 ± 4.76

“W” stands for the Western medicine treatment group; “J” stands for the JQF treatment group.

Comparison of women with dysmenorrhea treated for the first and third cycles and the follow-up group, **P < 0.01, *P < 0.05.

Comparison of women with dysmenorrhea with women in the volunteer group, before treatment ^##^ *P* < 0.01, ^#^ *P* < 0.05.

**Table 4:** E_2_, P, PGF_2*α*_, and PGE_2_ levels of dysmenorrhea rat uterine tissue in different groups (*χ* ± *S*, *n* = 8, ng/mL).

	E_2_	P	PGF_2*α*_	PGE_2_	PGF_2*α*_/PGE_2_
Control	19.07 ± 3.32	36.20 ± 24.76	209.65 ± 77.51	801.12 ± 111.32	0.27 ± 0.11
Model group	38.75 ± 10.57**	18.02 ± 15.01*	802.79 ± 153.69**	594.88 ± 137.15**	1.50 ± 0.56**
J one cycle	23.63 ± 3.09^##^	23.11 ± 8.96^#^	505.65 ± 98.02^##^	619.65 ± 187.78	0.99 ± 0.39
J three cycles	21.43 ± 3.85^##^	28.25 ± 2.81^##^	221.42 ± 140.67^##^	810.35 ± 208.62^#^	0.27 ± 0.16^##^
W one cycle	25.94 ± 7.31^##^	20.02 ± 4.51	521.67 ± 31.31^##^	608.17 ± 23.81	0.85 ± 1.31
W three cycles	23.13 ± 3.09^##^	22.00 ± 3.38^#^	488.78 ± 134.22^##^	875.62 ± 244.11^#^	0.59 ± 0.12^##^

Note: compared with normal control group: **P < 0.01, compared with model group: ^##^ *P* < 0.01, ^#^ *P* < 0.05. *P < 0.05.
