# Supplementary material for: Effect of Jingqian Zhitong Fang on Serum Sex Hormone Levels in Women with Primary Dysmenorrhea
Source: Evid Based Complement Alternat Med. 2014 Apr 16;2014:876431. doi: 10.1155/2014/876431 (PMC4009195; doi:10.1155/2014/876431)
Supplement: Supplementary file 1 — JQF quantitative analysis was performed using high performance liquid chromatography-triple quadrupole mass spectrometry (HPLC-QqQ-MS) in positive and negative ion modes. The separation was performed on an Eclipse plus C18, 4.6∗10 mm, 3.5 um, by gradient elution using 0.1% formic acid in water (mobile phase A) and 0.1% formic acid in acetonitrile as mobile phases. 13 peaks (stachydrine, gallic acid, oxypaeoniflorin, chlorogenic acid, rutin, paeoniflorin, caffeic acid, vanillic acid, ferulic acid, benzoyl paeoniflorin, senkyunolide A, and ligustilide) in the HPLC-MS chromatograms were unequivocally identified by comparison of their retention times (RTs), molecular weights, and MS data with reference data from the literature. Compound concentrations and percentages of ingredients in JQF are listed in Table S2 and Table S3. [file 876431.f1.doc]

Qualitative analysis of the chemical composition of JQF

1. Experimental

1.1 Reagents and materials

JQF components, including Angelica sinensis, Herba Leonuri, Pollen Typhae Angustifoliae, Rhizoma chuanxiong, Faeces Trogopterpri, Radix paeoniae Rubra, Ramulus Cinnamomi, Atractylodes macrocephala Koidz, Wolfiporia cocos, Carthamus tinctorius L., and peach kernel, were purchased from Tianjin Zhongxin Pharmaceutical Company Co., Ltd. The drugs were confirmed by the Traditional Chinese Medicine Plants Laboratory of Tianjin University. To avoid possible biases resulting from differences in herbs produced in different areas, all of the herbs were purchased at the same time and from the same area.

All standard substances were purchased from the National Institute for the Control of Pharmaceutical and Biological Products (Tianjin, China). The purity of each compound was confirmed to be higher than 98% by HPLC. Each reference compound was accurately weighed and dissolved in methanol to yield stock solutions.

HPLC-grade acetonitrile was purchased from Sigma (USA). The methanol and formic acid used were of analytical grade (Tianjin Chemical Reagent Co.)

1.2 Preparation of standard solutions

All standard substances were prepared in methanol at a concentration of 50 ng/mL and stored in the refrigerator at 4 ℃. Solutions were brought to room temperature before use.

1.3 Preparation of sample solutions

JQF medication was prepared as follows: The herbs Angelica sinensis, Herba Leonuri, Pollen Typhae Angustifoliae, Rhizoma chuanxiong, Faeces Trogopterpri, Radix paeoniae Rubra, Ramulus Cinnamomi, Atractylodes macrocephala Koidz, Wolfiporia cocos, Carthamus tinctorius L., and peach kernel, were mixed together at a ratio of 6:6:3:2:3:2:2:2:2:2:2 with water amounting to 10 times the volume of the herbs, and the resultant mixture was boiled for 1 h. The first extract was poured into a holding vessel. The same volume of water was added to the residue, and the mixture was boiled once more for another hour. The second extract was poured into the vessel containing the first extract, and the two extracts were mixed.

Exactly 10 mL of the aqueous solution of medicinal herbs was placed into a centrifuge tube and then centrifuged at 10,000 rpm and 4 °C for 10 min. The supernatant was filtered through a 0.45 μm membrane and then stored at –20 °C until analysis.

2. HPLC /MS analysis

HPLC/MS analysis was performed using a liquid mass spectrometry system (Applied Biosystems, USA). Ten microliters of the supernatant was injected onto an Eclipse plusC18, 4.6*10 mm, 3.5 um, Agilent. Here, the column temperature was set to 45 °C, and the flow rate was controlled to 0.4 mL/min. The gradient system consisted of 0.1% formic acid in water (mobile phase A) and 0.1% formic acid in acetonitrile (mobile phase B): 0–0.01 min, 90% A; 0.01–2 min, 90% A; 2–3 min, 90%–70% A; 3–5 min, 70%–15% A; 5–9 min, 15%-0% A; 9–13 min, 0% A ; 13–13.01 min, 0-90% A. and 13.01–16 min, 90% A. MS was equipped with an electrospray ionization (ESI) source. Scans were performed in both positive and negative ionization mode.

3. Results

Most of the authentic compounds exhibited [M-H]^-^ and [M+H]^+^ ions of sufficient abundance; these ions were subjected to MS/MS analysis and utilized for the structural identification of compounds with similar fragmentation patterns. 13 peaks in the HPLC-MS chromatograms were unequivocally identified by comparison of their retention times (RTs), molecular weights, and MS data with reference data from the literature.[1-9] Table S2 and Table S3 lists the RTs, compound concentrations and percentages of ingredients in JQF. Chromatograms in positive and negative ion mode are presented in Figure 1 and Figure 2 .

4. Conclusion

Ligustilide is the main ingredient in volatile oil from angelica. Ligustilide can ease the effects of smooth muscle spasms in both normal non-pregnant and oxytocin-treated uteri. [10]Stachydrine is the main component of motherwort. Chuanxiong can affect the blood circulation of qi and cools blood, thereby imparting analgesic efficacy; it is often used in the clinical treatment of irregular menstruation, amenorrhea dysmenorrhea, and headache. The main components of chuanxiong include volatile oils, alkaloids, phenolic substances, and organic acids. Ferulic acid, tetramethylpyrazine, and ligustilide exert antispasmodic effects on smooth muscles and can lead to uterine smooth muscle relaxation.[11-12] Radix paeoniae Rubra is rich in glycosides; the herb can heat or cool blood, heal bruises, and relieve pain. [13]Active ingredients associated with the therapeutic effects of JQF aqueous solution on dysmenorrhea may be extracted from motherwort, Chuanxiong, Radix paeoniae Rubra, and angelica.

Reference

1.Influence of different solutions on extraction of active components in Danggui Chishao drug pair. Ding Wen, Qian Dawei, Liu Pei, et.al. China Journal of Chinese Materia Medica 2012; 37(7): 916-9.

2.Analysis on chemical components from water extract of ligusticum chuanxiong by UPLC-ESI-Q-TOF-MS. Xu Xiao-fang, Sun Dong-dong, Li Xiang. Journal of Nanjing university of TCM 2013; 29(4):382-6.

3.Isolation and identification of constituents from Leonurus japonicus. Zhang Yi, Deng Shen, Li Xiao-xia. Chinese Journal of Medicinal Chemistry 2013;23(6):480-5.

4.Study on the active constituents in “Xue-Fu- Zhu-Yu capsule” and quantitative analysis of steroid saponins in Rhizoma Paridis. Ma Chao-yi. Tianjin University.

# 5.Identification and determination ofthe major constituents in Traditional Chinese

# Medicinal formula Danggui-Shaoyao-San by HPLC-DAD-ESI-MS/MS. Linlin Chen, Jin Qi, Yan-xu, Chang.J Pharm Biomed Anal 2009; 50(2): 127-37.

# 6.High-performance liquid chromatography with atmospheric pressure chemical ioniation and electrosprayionization mass spectrometry for analysis of Angelica sinensis.Wang YL. Liang YZ, Chen BM. Phytochem Anal 2007;18(4):265-74.

# 7.Chemical fingerprinting and quantitative constituent analysis of Siwu decoction categorized formulae by UPLC-QTOF/MS/MS and HPLC-DAD. Su S, Cui W, Zhou W. Chin Med 2013;8(1):5.

# 8. A high performance liquid chromatography fingerprinting and ultra high performance liquid chromatography coupled with quadrupole time-of- flight mass soectrometry chemical profiling approach to rapidly fing characteristic chemical markers for quality evaluation of dispensing granules, a case study on Chuanxiong Rizoma. Zhang XL, Liu LF, Zhu LY. J Pharm Biomed Anal 2014;88:391-400.

# 9.Characterization and quantification of major constituents of Xue Fu Zhu Yu by UPLC-DAD-MS/MS. Zhang L, Zhu L, Wang Y. J Pharm Biomed Anal 2012; 62:203-9.

10. Study Progress on Pharmacodynamics of DangGui and Its Active Ingredients. Ren Peng-fei, Deng Yi, Western Journal of Traditional Chinese Medicine 2012; 25(9):125-128.

11.Advances in modern medicine pharmacology Chuanxiong. Guo Jiansheng, Guo Qianyao. 239-243.

12.Progress of Chuanxiong volatile oil. Xie Xiuiqiong, Zhan Ke, Yin Rongli. LISHIZHEN Medicine And Materia Medica Research 2007; 18(6):1508-10.

13.Review in research of Radix Paeoniae Rubra.Ji Lan-xin,Huang Hao, Li Chang-zhi. Drug Evaluation Research. 2010 June 33(6) .233-236.

Table S2.Chemical analysis of JQF

| No | RT/min | Target compound | Mode | Prec ion(m/z) | Prod ion(m/z) | DP (V) | CE(eV) | CXP |
| --- | --- | --- | --- | --- | --- | --- | --- | --- |
|
| 1 | 2.52 | stachydrine | ESI+ | 144.064 | 84.000 | 101 | 33 | 14 |
| 2 | 4.62 | Gallic acid | ESI- | 168.713 | 124.800 | -75 | -22 | -7 |
| 3 | 6.83 | Oxypaeoniflorin | ESI- | 495.165 | 136.600 | -130 | -42 | -9 |
| 4 | 6.90 | Chlorogenic acid | ESI- | 353.070 | 190.900 | -70 | -22 | -8 |
| 5 | 6.99 | rutin | ESI- | 609.179 | 299.600 | -170 | -52 | -13 |
| 7 | 7.00 | Paeoniflorin | ESI- | 525.162 | 120.800 | -80 | -42 | -5 |
| 8 | 7.10 | Caffeic acid | ESI- | 178.893 | 134.800 | -75 | -24 | -9 |
| 9 | 7.16 | Vanillic acid | ESI- | 166.795 | 151.700 | -70 | -20 | -11 |
| 10 | 7.36 | Ferulic acid | ESI- | 192.854 | 134.800 | -65 | -20 | -9 |
| 11 | 7.54 | Benzoylpaeoniflorin | ESI- | 583.165 | 120.700 | -110 | -32 | -7 |
| 12 | 9.26 | Senkyunolide A | ESI+ | 193.100 | 91.400 | 81 | 35 | 15 |
| 13 | 9.86 | ligustilide | ESI+ | 191.044 | 173.200 | 101 | 23 | 10 |

Table S3.Quantitative analysis of JQF

| No | Ttarget compound | Standard curve | Concentration of the compound(ug/ml) | Percentage of the medicinal(%) |
| --- | --- | --- | --- | --- |
|
| 1 | stachydrine | y=3.68e+0.04x(r=0.9983) | 127 | 0.196 |
| 2 | Gallic acid | y=9.18e+0.03x(r=1.0000) | 20.5 | 0.032 |
| 3 | Oxypaeoniflorin | y=9.18e+0.03x(r=1.0000) | 39 | 0.060 |
| 4 | Chlorogenic acid | y=1.04e+0.04x(r=0.9997) | 13.05 | 0.020 |
| 5 | rutin | y=6.06e+0.03x(r=0.9998) | 6.595 | 0.010 |
| 7 | Paeoniflorin | y=6.59e+0.03x(r=0.9978) | 243 | 0.375 |
| 8 | Caffeic acid | y=2.4e+0.04x(r=0.9995) | 2.72 | 0.0042 |
| 9 | Vanillic acid | y=653x(r=0.9999) | 5.355 | 0.008 |
| 10 | Ferulic acid | y=5.93e+0.03x(r=0.9998) | 27.45 | 0.042 |
| 11 | Benzoylpaeoniflorin | y=3.49e+0.03x(r=0.9996) | 4.63 | 0.0072 |
| 12 | Senkyunolide A | y=3.75e+0.03x(r=0.9999) | 4.695 | 0.0073 |
| 13 | ligustilide | y=4.71e+0.05x(r=0.9970) | 0.00862 | 0.00001 |


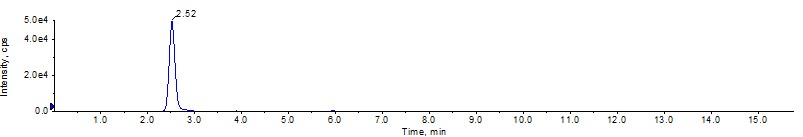


A

B


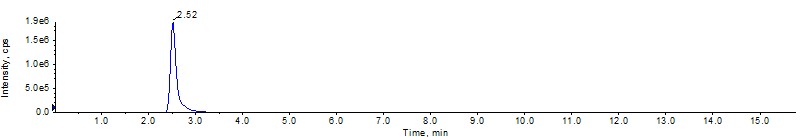

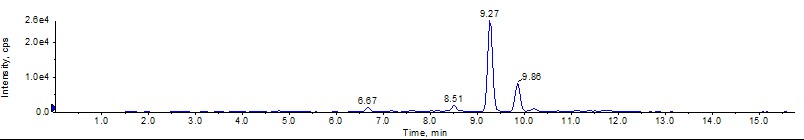

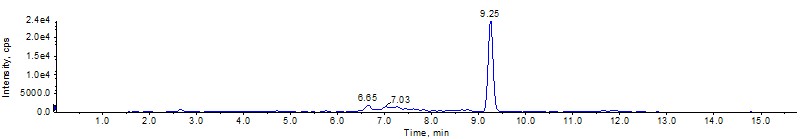

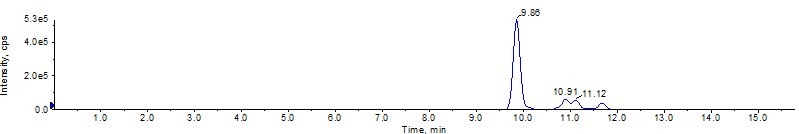

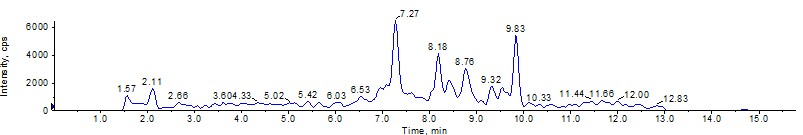


C

D

E

F

Figure S1. the standard solution and the JQF chromatograms of stachydrine (A, B) ,senkyunolide A (C, D), ligustilide (E, F) in positive ions.

A


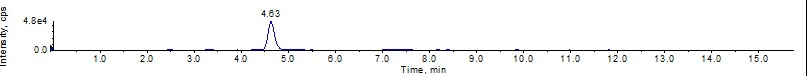


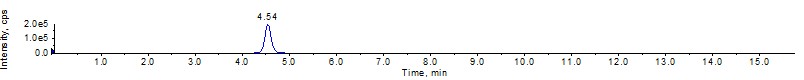


B


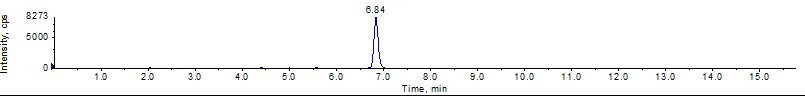

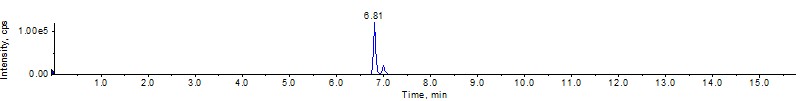


C

D

E


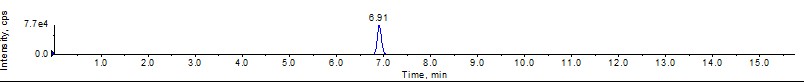

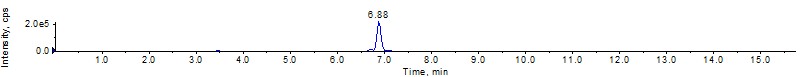


F


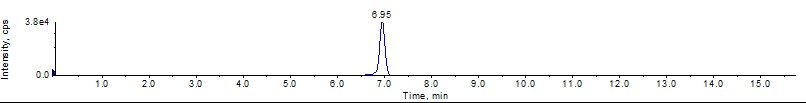

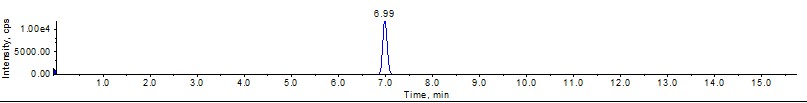


G

H


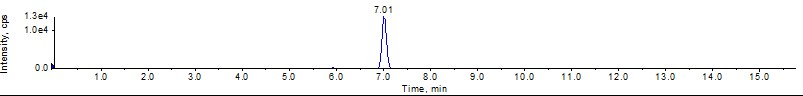

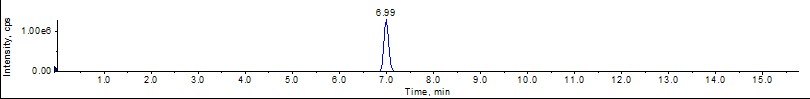


I

J

K


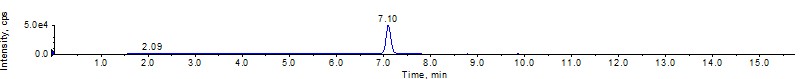


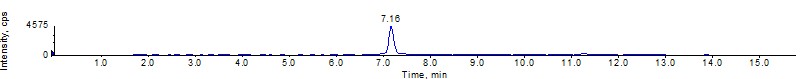

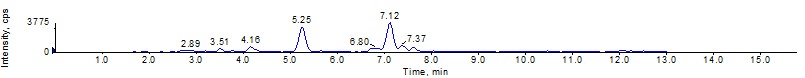

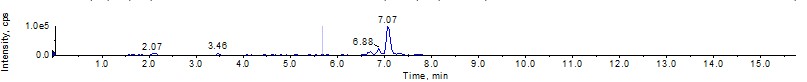


L

M

N

O


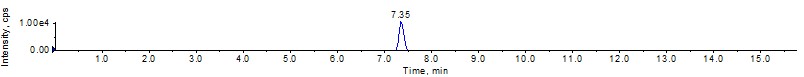

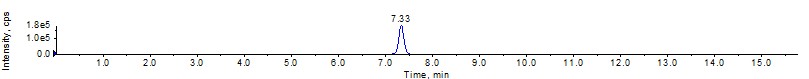


P


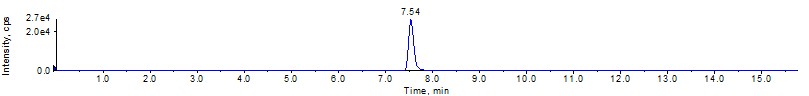

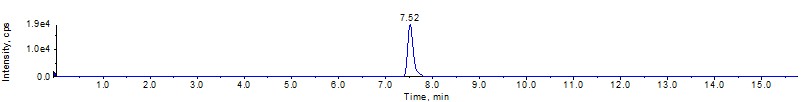


Q

R

Figure S2.The standard solution and the JQF chromatograms of gallic acid (A, B), oxypaeoniflorin(C, D), chlorogenic acid(E, F), rutin(G, H), paeoniflorin(I, J), caffeic acid(K, L), vanillic acid (M, N),ferulic acid(O, P), benzoylpaeoniflorin(Q, R) in negative ions.
